# Fracture Fixation Technique and Chewing Side Impact Jaw Mechanics in Mandible Fracture Repair

**DOI:** 10.1002/jbm4.10559

**Published:** 2021-10-13

**Authors:** Hyab Mehari Abraha, José Iriarte‐Diaz, Russell R Reid, Callum F Ross, Olga Panagiotopoulou

**Affiliations:** ^1^ Monash Biomedicine Discovery Institute, Department of Anatomy and Developmental Biology Monash University Melbourne Australia; ^2^ Department of Biology The University of the South Sewanee TN USA; ^3^ Department of Surgery, Section of Plastic Surgery The University of Chicago Medical Centre Chicago IL USA; ^4^ Department of Organismal Biology and Anatomy University of Chicago Chicago IL USA

**Keywords:** FINITE ELEMENT ANALYSIS, IMPLANTS, MANDIBLE, MASTICATION, MAXILLOFACIAL SURGERY, RHESUS MONKEY, TRAUMA

## Abstract

Lower jaw (mandible) fractures significantly impact patient health and well‐being due to pain and difficulty eating, but the best technique for repairing the most common subtype—angle fractures—and rehabilitating mastication is unknown. Our study is the first to use realistic in silico simulation of chewing to quantify the effects of Champy and biplanar techniques of angle fracture fixation. We show that more rigid, biplanar fixation results in lower strain magnitudes in the miniplates, the bone around the screws, and in the fracture zone, and that the mandibular strain regime approximates the unfractured condition. Importantly, the strain regime in the fracture zone is affected by chewing laterality, suggesting that both fixation type and the patient's post‐fixation masticatory pattern—ipsi‐ or contralateral to the fracture— impact the bone healing environment. Our study calls for further investigation of the impact of fixation technique on chewing behavior. Research that combines in vivo and in silico approaches can link jaw mechanics to bone healing and yield more definitive recommendations for fixation, hardware, and postoperative rehabilitation to improve outcomes. © 2021 The Authors. *JBMR Plus* published by Wiley Periodicals LLC on behalf of American Society for Bone and Mineral Research.

## 1 Introduction

Mandible fractures have many causes, including congenital disorders, oropharyngeal cancers, falls,^(^ ^1^, ^2^, ^3^ ^)^ battlefield injuries,^(^ ^4^, ^5^ ^)^ and vehicular accidents.^(^ ^6^, ^7^ ^)^ However, the single largest cause is interpersonal violence, mainly in young males who, in the US and Australia, are disproportionately members of minority populations.^(^ ^8^, ^9^ ^)^ Associated musculoskeletal disorders are a major cause of morbidity, with estimated hospitalization costs in the US of >$5 billion^(^ ^10^ ^)^ and further loss of quality of life and time off work during recovery.^(^ ^11^ ^)^ Treatment of mandible fractures aims to eliminate pain, promote bone healing, restore dental occlusion and jaw function, and improve facial aesthetics.^(^ ^2^, ^3^, ^12^ ^)^

**Fig. 1:**
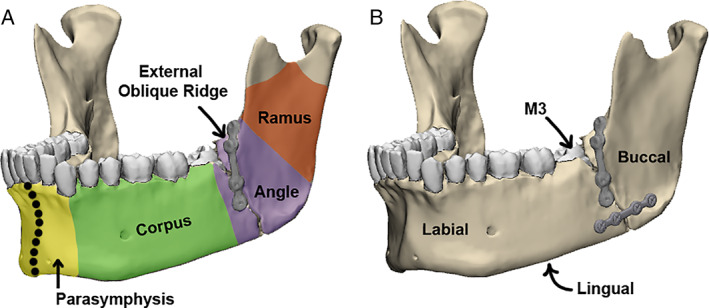
Terminology. (*A*) Single‐plate (Champy) fixation. (*B*) Biplanar fixation. The angle of the mandible (in purple) is at the junction between the ramus (in red) and corpus (in green).The symphysis is indicated by the dotted line and bordered on either side by the parasymphysis (in yellow). Labial (close to lip), lingual (close to tongue), and buccal (close to cheek) surfaces are indicated in black.

The most common mandible fracture site in adults is the angle region, extending from the corpus below the third molar (M_3_) to the angle at the back of the mandible (Fig. 1).^(^ ^7^ ^)^ Clinical treatment of angle fractures usually involves open reduction and internal fixation (ORIF) using either one or two miniplates.^(^ ^12^ ^)^ Single‐plate ORIF typically involves the Champy method: placement of one miniplate at the external oblique ridge, accessed transorally (Fig. 1*A*).^(^ ^12^, ^13^ ^)^ Champy fixation is less rigid, allowing some motion at the fracture line, especially inferiorly, which is thought to promote indirect bone healing, i.e., callus formation followed by secondary bone formation and remodeling.^(^ ^14^ ^)^ In contrast, more rigid ORIF of angle fractures involves two‐miniplate, biplanar fixation in which the Champy method is augmented by the placement of a second miniplate on the inferior lateral surface of the mandible, accessed through a transbuccal incision (through the cheek) with damage to the masseter muscle (Fig. 1*B*).^(^ ^12^, ^15^ ^)^ Biplanar fixation is more rigid, limiting micro‐motion between bone fragments, but is thought to promote direct bone healing, i.e., direct connection and healing of bone fragments by osteoblastic and osteoclastic activity but without callus formation.^(^ ^3^, ^14^ ^)^

There is an ongoing debate about whether Champy or biplanar fixation is the best treatment method for fixing angle fractures. The transoral approach used in Champy fixation is the least invasive and least disruptive to the masseter muscle and is often argued to be associated with fewer complications^(^ ^16^, ^17^, ^18^ ^)^ (but not always^(^ ^19^, ^20^ ^)^). Champy fixation is also assumed to result in more interfragmentary displacement (IFD), defined as the percentage change in interfragmentary distance, at the lower mandibular margin,^(^ ^21^, ^22^, ^23^ ^)^ but it is unknown whether this movement is the right amount, yielding optimal healing times and fewer mal/non‐unions, or too much, producing more mal/non‐unions. Biplanar fixation certainly offers better mechanical stability than single‐plate fixation,^(^ ^21^, ^24^ ^)^ but it is unknown whether it *over*‐stabilizes the fracture, reducing strain in the fracture line and inhibiting healing. It is also unclear which of these techniques best recovers the prefracture strain environment of the angle region, promoting fracture healing and ensuring mandibular function.

The rhesus macaque serves as an excellent animal model to study the effect of different fixation techniques on the strain environment in the mandible. Our team has previously used a combination of in vivo experiments and computational modeling to study the biomechanics of the jaw in healthy rhesus macaques.^(^ ^25^, ^26^, ^27^, ^28^, ^29^, ^30^ ^)^ We collected in vivo data on muscle activation, bone strain, and three‐dimensional (3D) jaw kinematics during chewing, combined these data with ex vivo measurements of bone material and muscle properties, and built a subject‐specific, validated finite element model (FEM) of the macaque jaw during unilateral post‐canine chewing.^(^ ^25^, ^26^, ^28^, ^29^ ^)^ We tested a series of hypotheses about loading regimes (the combination of external forces acting on the mandible), deformation regimes (the change in mandible shape), and stress and strain regimes (patterns of internal forces and strains associated with loading and deformation regimes) in the unfractured mandible, including the corpus‐ramus junction—the region where angle fractures occur.^(^ ^26^ ^)^ As in humans,^(^ ^31^, ^32^ ^)^ during simulated unilateral chewing in healthy macaques, the balancing (non‐chewing) side mandibular corpus is twisted about its long axis such that the alveolar process is inverted, negatively bent (concave inferiorly), and negatively sheared in sagittal planes.^(^ ^28^ ^)^ On the working (chewing) side, the posterior corpus is twisted about its long axis such that the alveolar process is everted, positively bent and negatively sheared in sagittal planes, and laterally bent in transverse planes.^(^ ^28^ ^)^ The macaque mandible's overall deformation pattern is remarkably similar to that of humans,^(^ ^28^, ^31^, ^32^, ^33^ ^)^ supporting the use of the macaque mandible as a model of human jaw function.

**Fig. 2:**
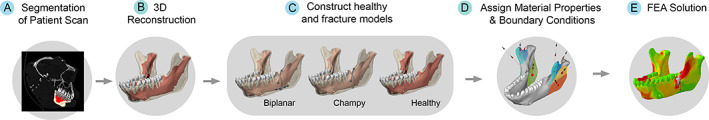
Flow chart of finite element analysis (FEA). (*A*) Patient‐specific computed tomography scans were processed to (*B*) create 3D models of the healthy controls and (*C*) the angle fracture fixation treatments. (*D*) All models were assigned the same tissue material properties and boundary conditions to simulate post‐canine chewing and (*E*) solved using Abaqus static implicit solvers.

In this study, we used this model of our healthy control to compare the impact of the Champy and biplanar angle fracture fixation techniques on the strain regime in the titanium plates, in the bone around the implant screws, in the bone around the fracture, and in the mandible globally. In silico, we simulated a fracture in the left angle, then repaired it with models of titanium 64‐alloy miniplates (26.1 mm × 2.5 mm × 1 mm) and bone screws using either the Champy or biplanar fixation techniques. The models were assigned the same tissue material properties, boundary conditions, and muscle forces as the healthy control, then loaded to simulate chewing ipsilateral and contralateral relative to the fracture (Fig. 2). We compared the effects of fixation technique on the moments acting on the mandible, as well as on strains in the titanium plates, the bone around the screws, the bone on either side of the fracture, the fracture gap itself, and the mandible more distant from the fracture.

With respect to Champy fixation, we hypothesized that (i) because Champy fixation concentrates the load path through a single plate, it would significantly alter the loading regime of the fractured mandible and be associated with higher principal strains at the bone‐implant interfaces; (ii) because it is the least rigid, it would be associated with the largest interfragmentary movement at the fracture gap;^(^ ^22^, ^24^, ^34^, ^35^ ^)^ and (iii) because the presence of the fracture redirects the load path through the implant construct, Champy fixation would reduce strains (strain shielding) around the fracture zone, particularly inferiorly.

With respect to biplanar fixation, we hypothesized that (i) because biplanar fixation transfers load through two plates, it would have less effect on the loading regime of the fractured mandible and would be associated with lower strains at the bone implant interfaces; (ii) because it is the most rigid fixation, it would be associated with the least interfragmentary movement; and (iii) because the presence of the fracture redirects the load path through the implant construct, biplanar fixation would result in strain reduction around the fracture zone both inferiorly and superiorly.

## 2 Materials and Methods

### 2.1 Macaque finite element models (FEMs)

#### 2.1.1 Healthy control

We modified our previously published and validated subject‐specific FEMs of healthy macaque chewing to simulate the two fracture fixation treatments (Champy and biplanar).^(^ ^26^, ^29^ ^)^

In brief, the 3D geometry of the macaque mandible and cranium was captured using computed tomography (CT) on a Philips Brilliance Big Bore scanner at the University of Chicago^(^ ^29^ ^)^ (Fig. 2*A*). The scans were processed in Mimics v17 software (Materialize, Leuven, Belgium) using manual and automatic segmentation methods to separate the cranium from the jaw and create 3D surface data of the mandibular tissues of interest (trabecular tissue, cortical bone, teeth, and anterior bone screws)^(^ ^29^ ^)^ (Fig. 2*B*). The periodontal ligament was excluded from our analyses because it does not substantially impact global strain regimes and increases computational time.^(^ ^26^ ^)^ The 3D surface files of the different tissues of the healthy control were imported into 3‐matic v15 (Materialize) to create a 3D non‐manifold assembly, converted into volumetric mesh files of linear tetrahedral elements, then imported into Abaqus CAE Simulia (Dassault Systémes, Vélicy‐Villacoublay, France) software for modeling. All mesh details are provided in Supplemental Table S1.

Isotropic, homogeneous, and linear elastic material properties were assigned to the teeth (E = 24.5 GPa; v = 0.3) and trabecular bone tissue (E = 10 GPa; v = 0.3). The cortical bone was modeled as heterogeneous and orthotropic using our published measurements of bone properties^(^ ^25^ ^)^ adjusted to the radiodensity of the calibrated CTs.^(^ ^25^, ^29^ ^)^ Tie constraints were used to bind together intersecting surfaces in the models to eliminate friction, known to influence FEA results.^(^ ^36^ ^)^

Experience suggests that realistic strain regimes (including lateral transverse bending) are obtained by fixing the working (chewing) side mandibular condyle against displacement in all directions and the balancing (non‐chewing) side condyle in anterior–posterior and superior–inferior directions only; the balancing condyle was allowed mediolateral translation to simulate lateral wish‐boning of the mandible during the power stroke of mastication. Bite forces resulted from constraining nodes on the occlusal surfaces of the chewing side premolars and first molar against all translations.^(^ ^26^, ^28^, ^29^ ^)^ Models were loaded using muscle activity data collected in vivo (Supplemental Data S1).^(^ ^29^ ^)^ To apply muscle forces, for each jaw muscle (anterior and posterior temporalis; deep and superficial masseters; medial pterygoids) surface nodes representing the origin and insertion were selected on the mandible and cranium, a directional vector joining the origin and insertion centroids was calculated, and these vector components were used to assign muscle force orientations at the mandibular insertion nodes.^(^ ^28^, ^29^ ^)^ Magnitudes of the muscle forces were estimated as the mean normalized EMG amplitude × estimated muscle physiological cross‐sectional area (PCSA) × specific tension of muscle (30 N/cm^2^).^(^ ^26^, ^28^, ^29^, ^37^ ^)^ PCSAs published in Panagiotopoulou and colleagues^(^ ^29^ ^)^ were used. FEM solution of all models was performed using the Abaqus direct implicit static solver.

#### 2.1.2 Treatment FEMs

To simulate angle fracture on the healthy control for the current study, we used 3‐Matic v15.0 and created a 0.2 mm planar cut in the angle of the left side mandible. The fracture line was then repaired with two different techniques, Champy and biplanar (Fig. 2*C*) fixation using 26.1 mm × 2.5 mm × 1 mm locking (threaded) miniplates with 4.2 mm self‐locking screws. We did not geometrically model a thread in the miniplate. Instead, we bound the internal surface of the screw head to the plate to mimic a thread. The components of each model (cortical bone, trabecular tissue, teeth, miniplates, and screws) were then collated into a non‐manifold assembly (Fig. 2*C*), converted to volumetric .inp files, and exported to Abaqus Simulia CAE 2016 for solution. All mesh files passed standard 3‐Matic and Abaqus 2016 mesh quality checks. All mesh details are provided in Supplemental Table S1. A close view of the 3D mesh of the bone interface and fracture zone of Champy and biplanar FEMs is provided in Supplemental Fig. S1. The mandible was assigned the same material properties as the healthy control (Fig. 2*D*). All implant materials (anterior bone screws, miniplates, fixation screws) were modeled as isotropic and homogeneous (E = 105 000 MPa; v = 0.36).^(^ ^29^, ^38^ ^)^

The interfaces between adjacent biological surfaces (cortical bone‐teeth, trabecular tissue‐teeth, trabecular tissue‐cortical bone) were modeled as tie constraints (surface‐to‐surface interaction with no relative motion). All screw‐bone surfaces were modeled as tie constraints (to replicate screw threads bonded to bone). The surface interactions between cortical bone, miniplates, and screws and between fracture segments were defined as “hard” contacts, with a penalty static friction coefficient of 0.3.^(^ ^35^, ^38^, ^39^, ^40^, ^42^ ^)^

Like the healthy control, all FEMs were solved using in Abaqus CAE default implicit direct static solver (Dassault Systémes) (Fig. 2*E*). Average solution time (six processors and eight tokens) was ∼30 minutes per model.

#### 2.1.3 Numerical comparison

When calculating the maximum strain values in the bone implant interface and in the implant tissues, we excluded nodes that reported strains outside two standard deviations from the mean (excluded top and bottom 5% of values). This was to ensure that elements with very low aspect ratios were excluded from any numerical comparison, as they report unrealistic strain results (~45,000 με) that are likely an artifact of the interface between cortical bone and the plate construct.

The resultant moments in Abaqus were calculated about coordinate axes through centroids of cross sections. As a result, the moments in each of the sections are moments about a point in the local coordinate system, given by the sum of all moments due to the internal forces acting at the section relative to the local coordinate system origin.

## 3 Results

**Fig. 3:**
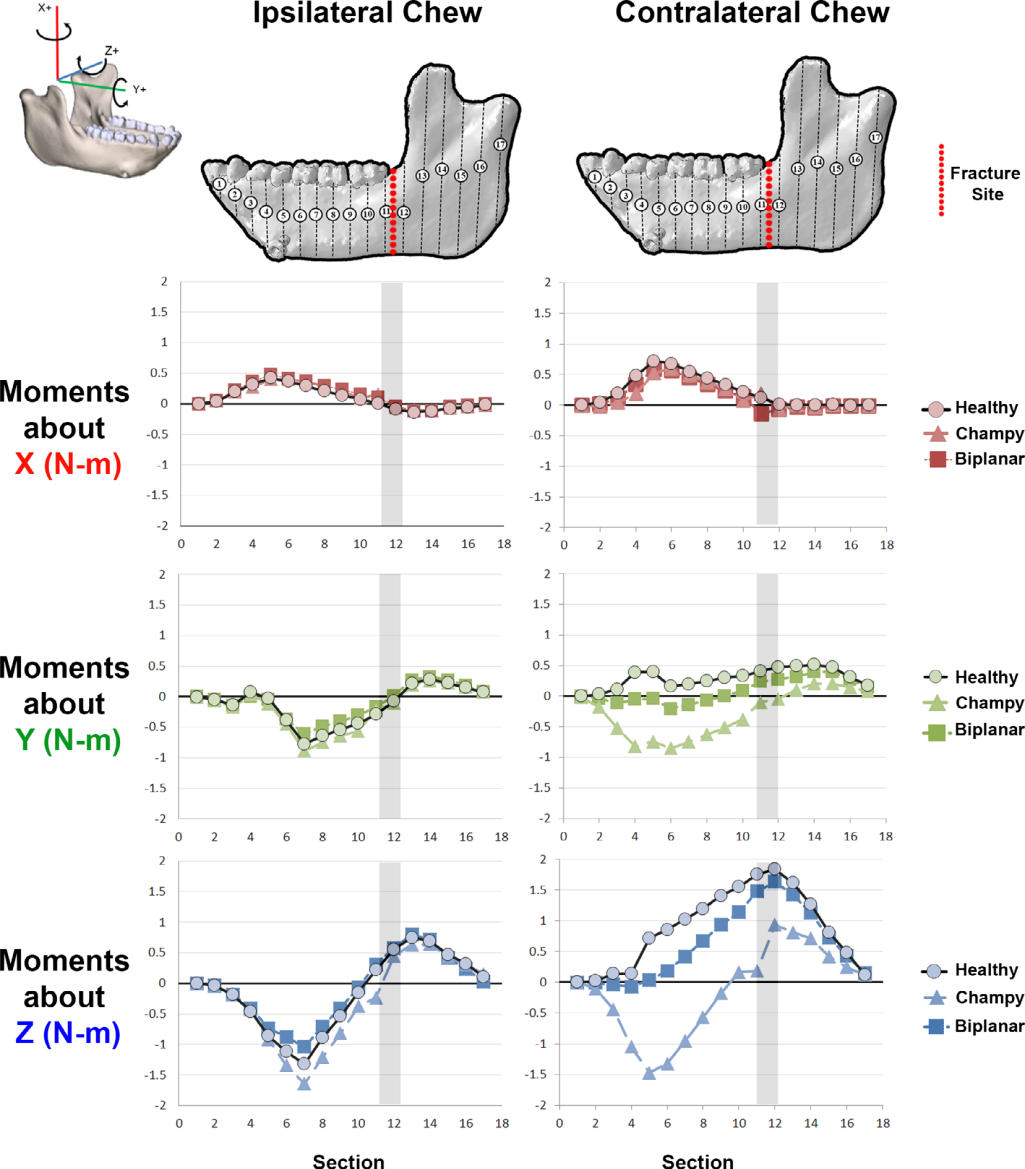
Moments (N‐m) acting about coronal sections through the mandible models during simulation of ipsilateral and contralateral chewing. Gray shading indicates fracture location.

In macaques (as in humans),^(^ ^28^ ^)^ the largest moments acting on the angle region are sagittal bending moments, and these are higher during contralateral chewing than in ipsilateral chewing (Fig. 3). Fracture fixation technique had little impact on the loading regime of the mandible during ipsilateral chews, but it greatly altered moments acting around the fracture plane during contralateral chews (Fig. 3; Supplementary Video S1). Champy fixation had the greatest effect, effectively eliminating anterior–posterior (AP) twisting moments (moments about Y) acting on the mandible immediately in front of and behind the fracture, eliminating sagittal bending moments just in front of the fracture, and halving sagittal bending moments just behind the fracture (Fig. 3). Moreover, the Champy technique also altered the loading regime along the entire length of the corpus between the fracture and the symphyseal region during contralateral chews (Fig. 3), changing the AP twisting moments from positive to negative and significantly increasing their magnitude (Fig. 3).

**Fig. 4:**
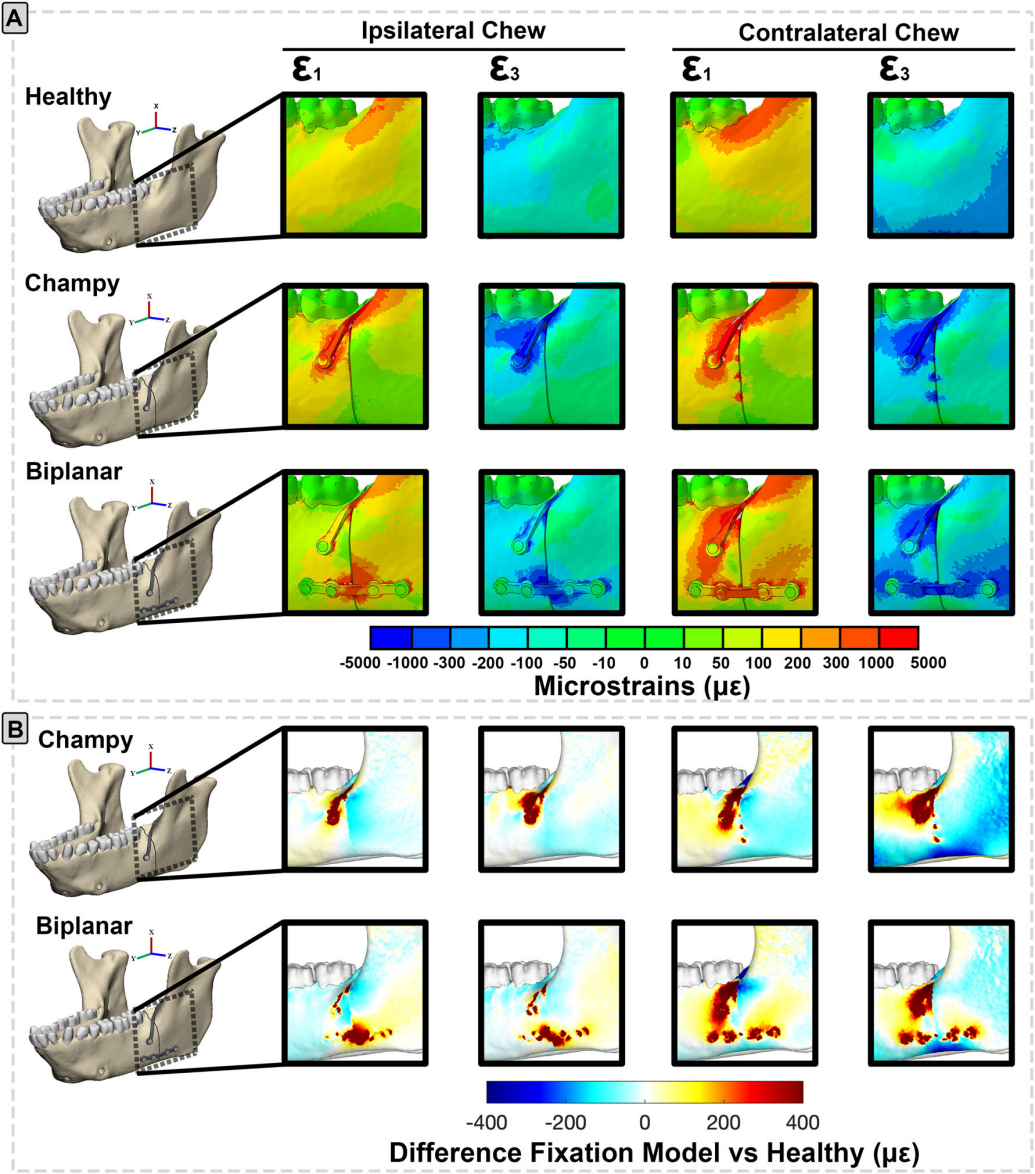
(*A*) Maximum (ε_1_‐positive values indicative of tension) and minimum (ε_3_‐negative values indicative of compression) principal strain in the bone implant interface and fracture zone of the angle fracture fixation treatments (Champy, biplanar) and in the healthy control during ipsi‐ and contralateral chews. Scale bar indicates strain magnitudes in microstrain. Warm colors = larger positive strain magnitudes. Cold colors = larger negative strain magnitudes. Green = low strain magnitudes. (*B*) Differences in maximum (ε_1_) and minimum (ε_3_) principal strain magnitudes between healthy control and fracture models repaired with Champy or biplanar technique during chews ipsilateral or contralateral to fracture. Plates and screws are not included in comparisons. Scale bar indicates difference in microstrain between fixed and healthy models. White = no difference in strain magnitudes. Warm colors = larger strains in fixed than control model. Cold colors = lower strains in fixed than control model. [Correction added on 16 December 2021, after first online publication: figure 4 has been replaced].

These changes to the loading regime are reflected in changes in the strain environment both in and around the implants and fracture and were strongly impacted by whether chewing occurred ipsilateral or contralateral to the fracture. As predicted, concentration of the load path through a single plate under Champy fixation resulted in higher principal strain magnitudes in the plates and in the bone‐screw interface than under biplanar fixation, especially under chewing contralateral to the fracture (Fig. 4*A*; Table 1). This concentration of the load path through the plates and screws was accompanied by significant strain shielding (lower ε_1_ and ε_3_ strain magnitudes compared with the healthy, blue colors in Fig. 4*B*) behind the fracture during ipsi‐ and contralateral chewing after Champy fixation, strain shielding in ε_1_ on either side of the upper half of the fracture and in both ε_1_ and ε_3_ on either side of the lower fracture during ipsi‐ and contralateral chewing after biplanar fixation (Fig. 4*B*). We also found very low von Mises stresses (<80 MPa) in the bone implant interfaces with high stresses (>150 MPa) only localized in the titanium plate. Miniplate von Mises stresses were the highest in the Champy fixation condition and during contralateral chews (Supplemental Fig. S2). Elsewhere around the fracture, both fixation techniques resulted in decreases in one principal strain magnitude (ε_1_) and increases in the other (ε_3_).

**Table 1:** Largest Maximum (ε1) and Minimum (ε3) Principal Strain Values (με) in the Bone Implant Interface and Plate Construction Under Different Mandibular Angle Fracture Fixation Techniques^a^

	Fixation	Bone implant interface		Plate construct	
		Largest ε_1_	Largest ε_3_	Largest ε_1_	Largest ε_3_
Ipsilateral chew	Biplanar	826	−31	227	−12
	Champy	2519	−83	1559	−25
Contralateral chew	Biplanar	2591	−54	749	−30
	Champy	5542	−203	2188	−70

^a^ Nodal strains in the top and bottom 5% were excluded because of potential modeling artifacts (e.g., interfacing surfaces).

**Fig. 5:**
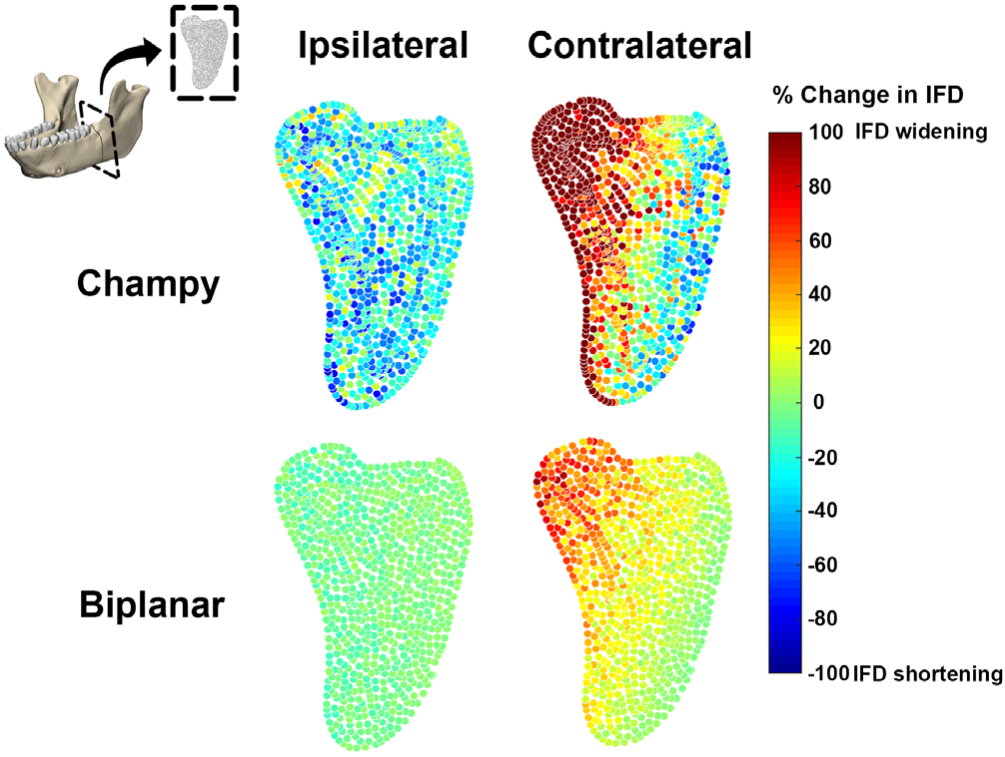
Percentage change in interfragmentary distance (IFD) between nodes across the fracture plane during chewing ipsi‐ and contralateral to the fracture in macaques. Warm and cold colors show areas with high and low IFD.

Within the fracture plane itself, there were marked differences in interfragmentary displacement between Champy and biplanar fixation and significant departures from traditional expectations (Fig. 5). Not surprisingly, the less rigid Champy fixation was associated with the greatest overall change in interfragmentary distance (Table 2), with an approximately 80% reduction in interfragmentary distance (IFD), indicative of almost uniform compression of the fracture during ipsilateral chewing. Contralateral chewing after Champy fixation was accompanied by an unexpected lateral bending of the fracture—tension medially (>80% fracture gap widening) and compression laterally (>80% gap distance shortening) (Fig. 5). As expected, the more rigid biplanar fixation resulted in a fracture zone that was essentially static (<10% change in IFD) during ipsilateral chewing, but the same lateral bending was found during contralateral chewing, albeit to a lesser degree than after Champy fixation (Fig. 5).

**Table 2:** Average (Mode) % Change in Interfragmentary Distance (IFD) for Different Mandibular Angle Fracture Fixation Finite Element Models Under Chewing Loads

	Fixation	No. of nodes	IFD
Ipsilateral chew	Biplanar	909	−6
	Champy	1131	−51
Contralateral chew	Biplanar	909	14.5
	Champy	1131	162

**Fig. 6:**
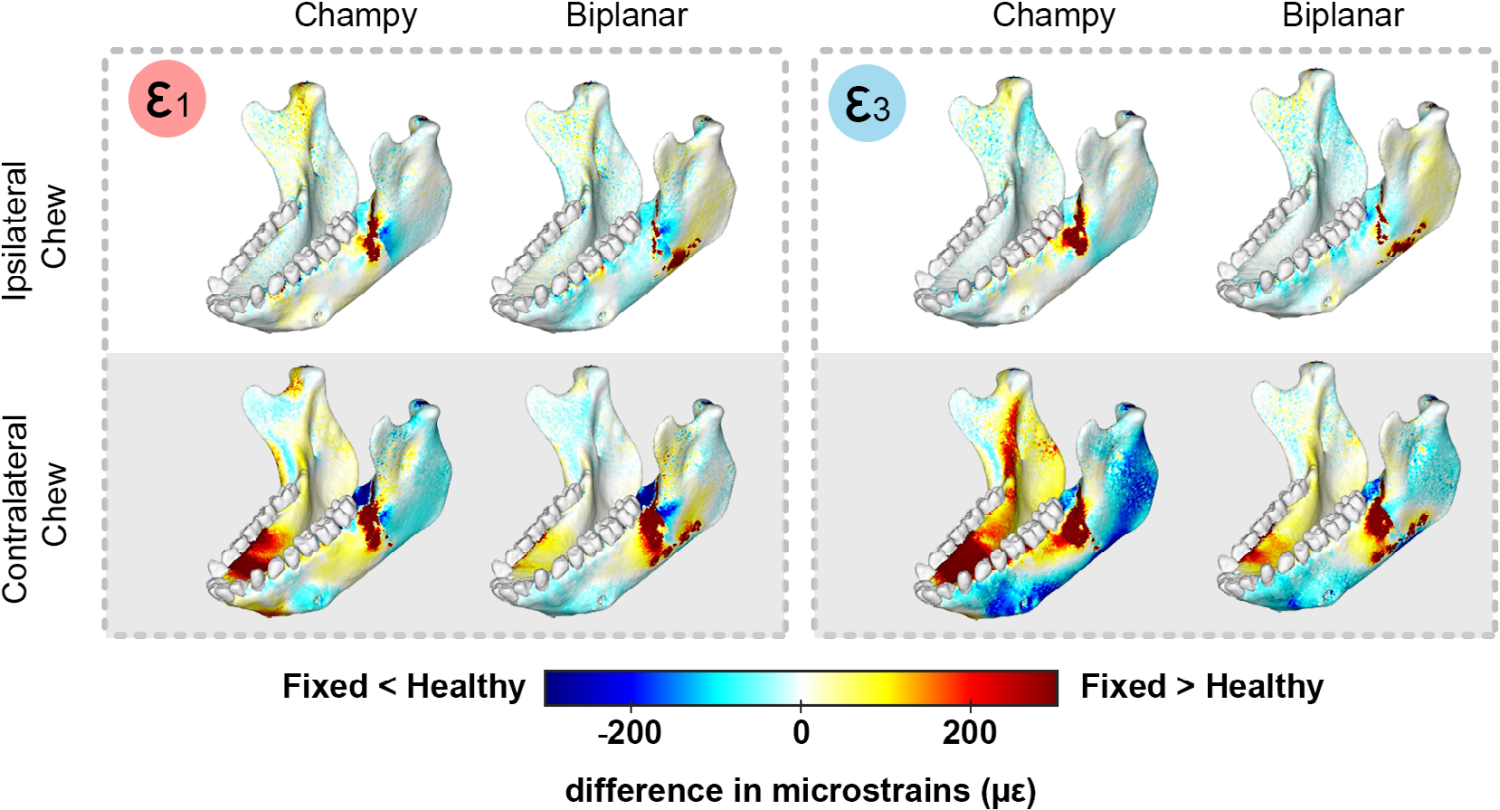
Differences in maximum (ε_1_) and minimum (ε_3_) principal strain magnitudes between healthy control and fracture models repaired with Champy or biplanar technique during chews ipsilateral or contralateral to the fracture. Plates and screws not included in these comparisons. Scale bar indicates difference in microstrain between healthy control and fracture models. White = no difference in strain magnitudes. Warm colors = larger strains in fixed than control. Cold colors = Lower strains in fixed than control.

Fixation technique also impacted strain regimes in the rest of the mandible, away from the fracture zone (Fig. 6). These effects were small during chewing ipsilateral to the fracture, with strains after both biplanar and Champy fixation deviating by <100 με from the healthy control. However, during chewing contralateral to the fracture, fixation technique significantly impacted strains away from the fracture zone and these effects were large (Fig. 6). In the Champy model, the greatest deviations, >400 με from healthy strains, were found in the labial and lingual faces of the right side parasymphysis (ipsilateral to the bite point and contralateral to the fracture) and in the lingual aspect of the left corpus (Fig. 6). In contrast, the biplanar fixation model only deviated substantially from the healthy control in the labial right parasymphysis, immediately below the loaded teeth (P3‐M1), and in a small area of the lingual left parasymphysis (Fig. 6).

## 4 Discussion

Our results show that the more rigid biplanar fixation technique is associated with the smallest changes in the loading regime acting around the fracture, the lowest strains in the titanium plates, the lowest strains in the bone‐implant interface, the least interfragmentary displacement, and a global mandibular strain regime that best matches the healthy control. This is especially true during contralateral chews, when the second lateral plate is well placed to resist the high sagittal bending moments acting across the fracture plane. Single‐plate Champy fixation results in higher strains in the plate, higher strains at the bone implant interface, high degrees of interfragmentary displacement, and global strains that deviate substantially from the healthy control, particularly during contralateral chews (Supplemental Video S1). Increased strains in the plate may be an artifact of the locking screws used. Clinically, in the majority of Champy plate fixations, the plates and screws are non‐locking. Because of lack of data on the coefficient of friction required to model the non‐locking screws, we optimized the plates and screws virtually so that they conform to the body surface and modeled the plate‐screws for the Champy fixation as locking. Future in vivo research will allow us to measure the friction coefficient at the bone implant interface experimentally and further optimize the modeling of the hardware for our treatments. Notably, Champy fixation leads to dramatic variations from healthy strains in areas far away from the fracture zone (e.g., right lingual parasymphysis). This finding suggests that the lack of rigidity after Champy fixation not only fails to stabilize the fracture plane but also results in changes to the global strain environment of the jaw.

Understanding the mechanical environment of the jaw post fracture and fixation is an essential prerequisite for designing repair and rehabilitative techniques that optimize patient outcomes.^(^ ^43^ ^)^ To understand how the mechanical environment of the jaw impacts healing, we first need to understand how different fixation techniques affect jaw mechanics.^(^ ^43^, ^44^ ^)^ This study used finite element analysis to test the impact of the two most clinically widespread angle fracture fixation techniques (Champy and biplanar) on strains in and around implants and the fracture zone during chewing. We found that because fracture fixation redirects the load path almost exclusively through the plate construct, the less rigid, single‐plate Champy fixation technique is associated with higher principal strains in the plate and the bone‐screw interface than the biplanar technique. This effect is exacerbated by chewing on the opposite side to the fracture, when sagittal bending moments acting on the fracture plane are greatest. Our results are consistent with those of Liu and colleagues,^(^ ^45^ ^)^ who found that single‐plate fixation is associated with higher stresses in the bone implant interface and the implant construct than two‐plate fixation.

If Champy fixation results in such a biomechanically unfavorable environment, why is it so successful clinically?^(^ ^1^ ^)^ The widely accepted explanation is that Champy fixation is less invasive than biplanar, especially in causing less disruption of masseter,^(^ ^1^, ^19^ ^)^ but how the two techniques affect muscle activity during feeding after fracture fixation is unknown. In this study, we used muscle loadings based on muscle recruitment patterns measured in healthy controls because the only available data on the impact of fixation technique on feeding system function are bite force data and EMG activity from easily accessible muscles during transducer biting.^(^ ^46^, ^47^, ^48^ ^)^ Data are urgently needed on post‐fracture jaw muscle activity and jaw kinematics during chewing to evaluate the potential for rehabilitative treatments to speed healing and functional recovery.

One of the most salient results of this study is the importance of the laterality of post‐fracture mastication. Our results show that contralateral chewing after Champy fixation is associated with much greater degrees of interfragmentary displacement (50% and 162%) than the optimal window of 10% suggested by orthopedic literature.^(^ ^49^, ^50^ ^)^ This suggests that mobile, less rigid fixation and contralateral chewing behavior may inhibit bone healing and contribute to the development of postoperative complications, such as non‐union or malunion of the fracture segments.^(^ ^51^ ^)^ In contrast, after either fixation technique, ipsilateral chewing generates strain regimes in the fracture zone that most closely resemble the healthy control. Hence, ipsilateral chewing using healthy control muscle recruitment patterns might speed healing success in *isolated* angle fractures. However, it is important to note that Champy fixation is accompanied by dramatically increased principal strains in the parasymphyseal region contralateral to the angle fracture, particularly during contralateral chews. This is exactly the location of the most common fracture occurring in combination with an angle fracture.^(^ ^52^, ^53^ ^)^ Hence, rehabilitation strategies for isolated angle fractures may actually be contra‐indicated for multiple fractures involving angle and parasymphseal regions. Clearly better data are needed on the impact of fracture fixation technique on muscle activity and mandibular loading and strain regimes.

Previous comparisons of angle fracture fixation techniques using benchtop experiments on cadaveric or resin‐based human mandibles have not replicated physiological loading conditions and have yielded conflicting results.^(^ ^21^, ^54^, ^55^ ^)^ By using a computer simulation environment and loading our finite element models with physiologically accurate muscle forces, our study is the first to simulate the strain environment in and around the fracture and implants during use of the jaw for its primary function—chewing. Thus our finite element models are the most robust testing environment for different angle fracture fixation techniques developed to date. Our results suggest that biplanar fixation should yield the best healing outcomes, but we hesitate to make firm recommendations until we can load our fracture‐fixed finite element models with muscle forces estimated using data from subjects with surgically repaired mandible fractures. Patients with repaired mandible fractures may change muscle force activation patterns consciously in response to pain or unconsciously in expectation of pain.^(^ ^11^, ^47^ ^)^ To our knowledge, no study has characterized the subject‐specific pre‐ and post‐fixation muscle activation patterns of all the major muscles of mastication during chewing. Incorporation of post‐fixation behavioral plasticity into models of the post‐fixation biomechanical environment of the jaw would significantly expand the translational reach of our modeling. Addressing these limitations and clarifying the relationship between fracture, treatment (fixation), behavioral plasticity, and healing are essential to the design of optimized treatment methods for angle fractures. Research integrating in vivo data with accurate in silico models offers the best opportunity to circumvent issues common with existing clinical research, such as variation in patient compliance, surgical expertise, and postoperative therapy. Only by combining in vivo experiments, biomechanical modeling, and histological analyses will we obtain a clearer understanding of the relationship between specific techniques of angle fracture fixation and bone healing in the mandible.

## Disclosures

All authors state that they have no conflicts of interest.

### Peer Review

The peer review history for this article is available at https://publons.com/publon/10.1002/jbm4.10559.

## Code Availability Statement

Custom MatLab and R code was written by Hyab Mehari Abraha to conduct interfragmentary distance and coefficient of static friction sensitivity analysis for all models. Code is available on Monash Bridges for analysis conducted in the main text and Supplemental Material on Monash Bridges at https://figshare.com/s/f7d8816fba6b72dc5f45 and https://figshare.com/s/20535cedf405e83707e6, respectively. Custom MatLab code was written by Jose Iriarte‐Diaz to conduct the whole model nodal comparisons of all models. Code is available at https://github.com/josdiiri/plotSurfaceStrains/.

## Supporting information


**Supplemental Data S1.** Muscle Force vectors used for all models

**Supplemental Fig. S1.** 3D mesh of the bone interface and fracture zone of the Champy and Biplanar FE models.

**Supplemental Fig. S2.** Von mises stress distribution at bone implant interface of all FEMs.

**Supplemental Table S1.** Supporting Information

**Supplemental Video S1.** Animated GIFs of 20x deformation of macaque finite element models (FEMs) plotted with principal (ε1 and ε3) strains. FEMs are of the mandible in the healthy (unfractured) and angle fracture fixed (Champy and biplanar fixation) conditions under masticatory loads.

## Data Availability

Submission (.inp) and results files (.odb) for all finite element models presented in the main text and in the Supplemental Material can be found on Monash Bridges at https://figshare.com/s/a46efafc1f4581db1001 and https://figshare.com/s/e942ed9d539f9e1ea74c, respectively. All muscle data used as part of this publication can be found in Supplemental Data S1. All data analysis files, including raw data from Abaqus (.rpt) and analysis code, for the main text and Supplemental Material can be found on Monash Bridges at https://figshare.com/s/f7d8816fba6b72dc5f45 and https://figshare.com/s/20535cedf405e83707e6, respectively. Data analysis files are organized by Figure and Table number.
