# Supplementary figures and images for: Fracture Fixation Technique and Chewing Side Impact Jaw Mechanics in Mandible Fracture Repair
Source: JBMR Plus. 2021 Oct 13;6(1):e10559. doi: 10.1002/jbm4.10559 (PMC8770999; doi:10.1002/jbm4.10559)

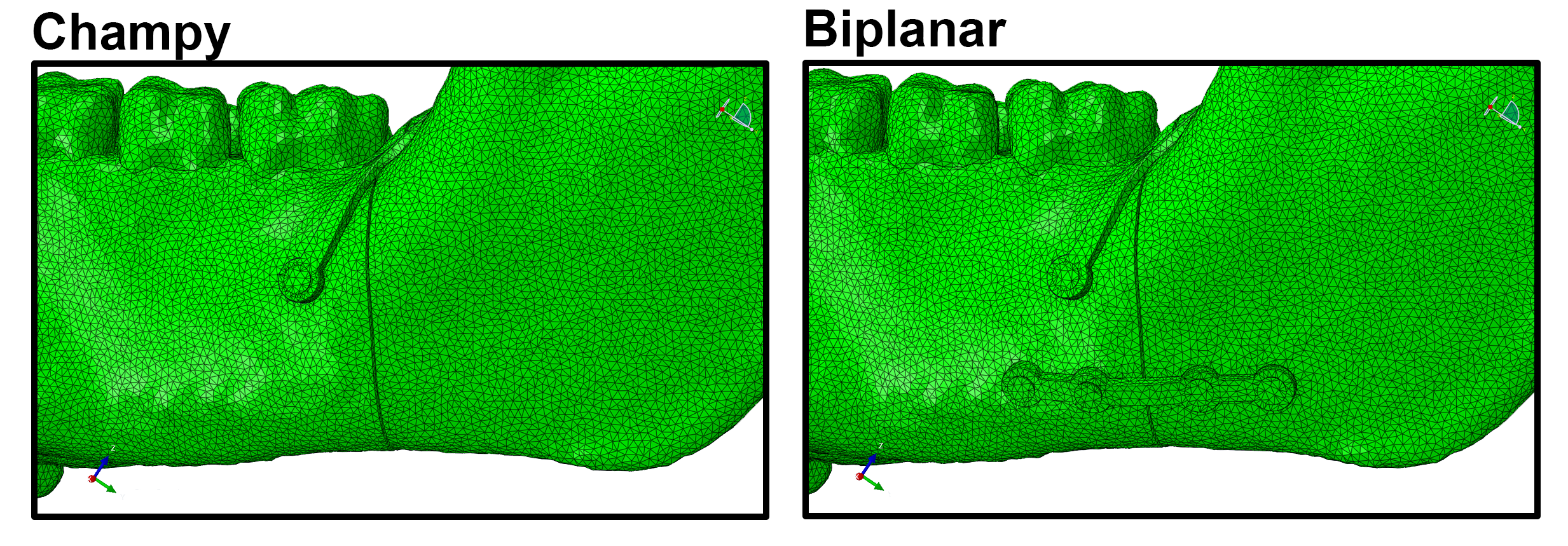

Supplement: Supplementary file 2 — Supplemental Fig. S1. 3D mesh of the bone interface and fracture zone of the Champy and Biplanar FE models. [file JBM4-6-e10559-s002.tif]

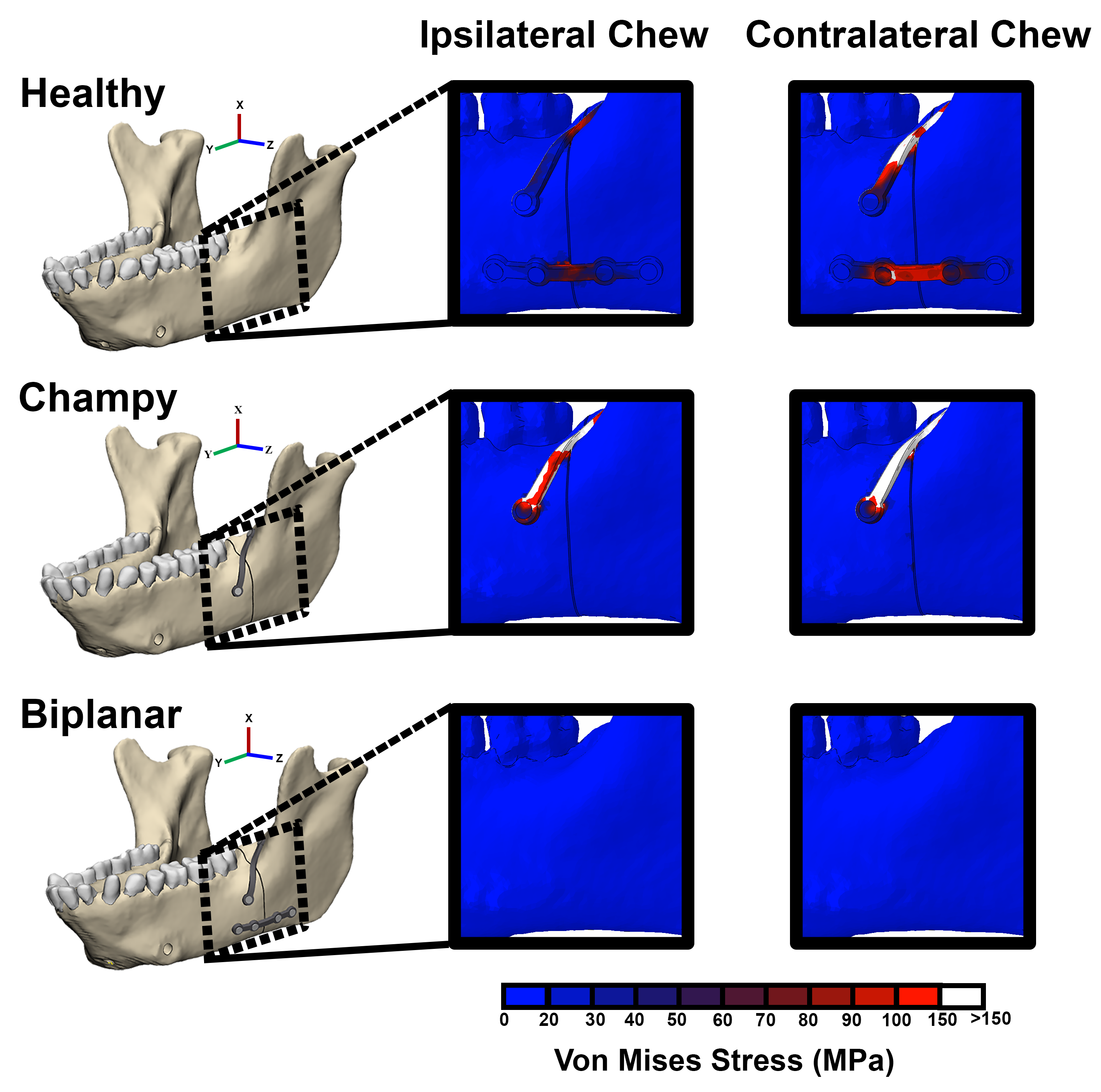

Supplement: Supplementary file 3 — Supplemental Fig. S2. Von mises stress distribution at bone implant interface of all FEMs. [file JBM4-6-e10559-s004.tif]
