# Supplementary material for: Fracture Fixation Technique and Chewing Side Impact Jaw Mechanics in Mandible Fracture Repair
Source: JBMR Plus. 2021 Oct 13;6(1):e10559. doi: 10.1002/jbm4.10559 (PMC8770999; doi:10.1002/jbm4.10559)
Supplement: Supplementary file 4 — Supplemental Table S1. Supporting Information [file JBM4-6-e10559-s005.docx]

Supplementary Table S1: Mesh specifications for all models.

| **Model** | **Volume (mm^2^)** | **No. of Element** | **No. of Vertices** | **Element Type** |
| --- | --- | --- | --- | --- |
| Healthy | 14530 | 622134 | 186006 | C3D4 |
| Champy | 14442 | 562666 | 163398 | C3D4 |
| Biplanar | 14417 | 647902 | 177726 | C3D4 |
